# Negative regulation of thyroid adenoma-associated protein (THADA) in the cardiac glycoside-induced anti-cancer effect

**DOI:** 10.1186/s12576-024-00914-7

**Published:** 2024-04-01

**Authors:** Mizuki Katoh, Takuto Fujii, Yoshiaki Tabuchi, Takahiro Shimizu, Hideki Sakai

**Affiliations:** 1 Department of Pharmaceutical Physiology, Faculty of Pharmaceutical Sciences, University of Toyama, Toyama, 930-0194 Japan; 2 Division of Molecular Genetics Research, Life Science Research Center, University of Toyama, Toyama, 930-0194 Japan

**Keywords:** Thyroid adenoma-associated protein, L-type amino acid transporter 1, Na^+^,K^+^-ATPase, Cardiac glycoside, Cancer cells

## Abstract

Cardiac glycosides, known as inhibitors of Na^+^,K^+^-ATPase, have anti-cancer effects such as suppression of cancer cell proliferation and induction of cancer cell death. Here, we examined the signaling pathway elicited by cardiac glycosides in the human hepatocellular carcinoma HepG2 cells and human epidermoid carcinoma KB cells. Three kinds of cardiac glycosides (ouabain, oleandrin, and digoxin) inhibited the cancer cell proliferation and decreased the expression level of thyroid adenoma-associated protein (THADA). Interestingly, the knockdown of THADA inhibited cancer cell proliferation, and the proliferation was significantly rescued by re-expression of THADA in the THADA-knockdown cells. In addition, the THADA-knockdown markedly decreased the expression level of L-type amino acid transporter LAT1. Cardiac glycosides also reduced the LAT1 expression. The LAT1 inhibitor, JPH203, significantly weakened the cancer cell proliferation. These results suggest that the binding of cardiac glycosides to Na^+^,K^+^-ATPase negatively regulates the THADA-LAT1 pathway, exerting the anti-proliferative effect in cancer cells.

**Supplementary Information:**

The online version contains supplementary material available at 10.1186/s12576-024-00914-7.

## Background

The gene of Thyroid adenoma associated (*THADA*) was identified as a target gene affected by chromosome 2p21 translocations in thyroid adenomas, and encodes the hypothetical 1954 amino acids (220 kDa) [1]. Thereafter, *THADA* was found to be associated with type 2 diabetes mellitus (T2DM) [2] and polycystic ovary syndrome (PCOS) [3].

Recently, several functions of the THADA protein have been reported. Moraru et al. [4] reported that THADA-knockout in flies (*Drosophila melanogaster*) led to obesity due to hyperphagia, reduced thermogenesis, and increased lipid storage. Interestingly, the THADA function requires interaction between THADA and the sarco/ER Ca^2+^-ATPase (SERCA) [4]. Zhang et al. [5] reported that THADA is strongly activated in human and mouse islets of T2DM. In the mechanism of impairment of insulin secretion in β-cells, activation of THADA decreases ER Ca^2+^ storage through SERCA2 and ryanodine receptor 2. In fact, THADA inhibition in mice protects against T2DM [5]. These findings suggest that function of THADA is associated with Ca^2+^-ATPase which belongs to P2-type ATPase family [6].

On the other hand, THADA may be involved in cancer pathogenesis. It has been reported that polymorphism of *THADA* may be associated with prostate cancer risk [7]. THADA is highly expressed in human colorectal cancer, and functions as an indispensable regulator of programmed death-ligand 1 (PD-L1) maturation [8].

So far, cardiac glycosides, such as ouabain, oleandrin, and digoxin, have been reported to suppress cell proliferation and induce cell death in cancer cells [9, 10]. Thus, Na^+^,K^+^-ATPase, a target of cardiac glycosides, is considered as a potent molecule with clinical benefit in cancer treatment [11]. Na^+^,K^+^-ATPase belongs to P2-type ATPase family as well as Ca^2+^-ATPase [6]. In the present study, we examined whether THADA contributes to the cardiac glycoside-induced anti-cancer mechanism and found that THADA is negatively regulated in the mechanism.

## Methods

### Chemicals

Ouabain, oleandrin, and digoxin were obtained from Sigma-Aldrich. JPH203 was from Selleckchem. Rabbit polyclonal anti-THADA antibody was from Bioworld Technology and Atlas Antibodies. Mouse monoclonal anti-4F2hc (Clone: E-5), mouse monoclonal anti-Na^+^,K^+^-ATPase α1-isoform (Clone: C464.6), and mouse monoclonal anti-Na^+^,K^+^-ATPase α3-isoform (Clone: XVIF9-G10) antibodies were from Santa Cruz Biotechnology. Mouse monoclonal anti-β-actin antibody (Clone: 8H10D10) was from Cell Signaling Technology. Mouse monoclonal anti-LAT1 antibody was from Trans Genic. Alexa Fluor 488- and 568-conjugated IgG antibodies were from Abcam. Lipofectamine 3000 was from Thermo Fisher Scientific. Screen Fect reagent was from Fujifilm Wako Pure Chemical. DAPI was from Dojindo Laboratories. THADA and negative control siRNA pools were from Dharmacon. All other reagents were of the molecular biological grade or the highest grade of purity available.

### Microarray gene expression analysis and RT-PCR

Microarray gene expression analysis was performed using a GeneChip system with a Human Genome U133-plus 2.0 array, which was spotted with 54,675 probe sets (Affymetrix, Inc.) according to the manufacturer's instructions. In brief, 500 ng of total RNA prepared from HepG2 cells was used to synthesize cRNA with a GeneChip 3′ IVT Express kit (Affymetrix, Inc.). Fragmentated biotin-labeled cRNA was hybridized to the array at 45 ℃ for 16 h. After the staining with streptavidin–phycoerythrin, the array was scanned using a probe array scanner. The obtained hybridization intensity data were analyzed using GeneSpring GX software (Agilent Technologies, Inc.) to extract the significant genes. In RT-PCR using total RNA of HepG2 cells, the following thermal conditions were used: a predenature of 94 °C for 30 s and the next 30 cycles of 94 °C for 30 s, 60 °C for 30 s and 68 °C for 19 s or 11 s. The designed primer pairs were as follows: for THADA: 5′-gaccatttgccatcagga-3′ and 5′-ggtgcatagcctcaggtaga-3′ and for glyceraldehyde-3-phosphate dehydrogenase (GAPDH): 5′-aacctgccaaatatgatgac-3′ and 5′-ataccaggaaatgagcttga-3′.

### Quantitative RT-PCR

Total RNA was extracted from the KB cells treated with or without ouabain (100 nM for 48 h) and transcribed into cDNA. Quantitative RT-PCR were conducted with Luna Universal qPCR Master Mix (New England Biolabs) in a real-time PCR system (Mx3000P: Agilent Technologies). The following thermal conditions were used: an initial denaturation of 95 °C for 60 s and the next 50 cycles of 95 °C for 15 s and 60 °C for 30 s. The designed primer pairs were as follows: for SLC2A13: 5′-cattgactcctcctgtgttcca-3′ and 5′-tcctgtacttcttgcccaaagg-3′, for SLC3A2: 5′-tctggttctactggggagcata-3′ and 5′-tctcatccccgtagctgaaaac-3′, for SLC7A5: 5′-cattatacagcggcctctttgc-3′ and 5′-caggtgatagttcccgaagtcc-3′, for SLC7A11: 5′-cttcatctctcctaagggcgtg-3′ and 5′-tccacccagactcgtacaaaag-3′, for SLC12A7: 5′-ttttctgacgtacatctccccg-3′ and 5′-cttgttgacatacttgacgccc-3′, for SLC39A9: 5′-ctggctatgttggtgggatgt-3′ and 5′-cttgcttggtggtgttttccc-3′ and for glyceraldehyde-3-phosphate dehydrogenase (GAPDH): 5′-aacctgccaaatatgatgac-3′ and 5′-ataccaggaaatgagcttga-3′.

### Cell culture

Human hepatocellular carcinoma HepG2 cells (RIKEN Cell Bank; RCB1648) and human epidermoid carcinoma KB cells (kindly gifted from Prof. Yasunobu Okada, National Institute for Physiological Sciences) were maintained in Minimum Essential Medium (MEM; Fujifilm Wako pure chemical) supplemented with 100 units/ml penicillin, 100 µg/ml streptomycin (Fujifilm Wako Pure Chemical), and 10% fetal bovine serum (FBS; Nichirei biosciences).

### Transfection of siRNA

KB cells (2 × 10^4^ cells) and HepG2 cells (1.5 × 10^5^ cells) were transfected with THADA siRNA (siTHADA) or negative control siRNAs (siNC) using Lipofectamine 3000 or Screen Fect reagent, and the medium was changed 5 h after transfection. The experiments were performed 48 h after the transfection unless otherwise described.

### Plasmid construction

A full-length cDNA encoding human THADA was cloned from the human colorectal cancer HT-29 cells. The fragment was inserted into the pcDNA4/His C vector by using BamHI and ApaI restriction sites. The cDNA sequence was verified using Big Dye Terminator V3.1 Cycle Sequencing Kit (Thermo Fisher Scientific) and an ABI PRISM 3500 sequencer (Applied Biosystems).

### Cell proliferation assay

To check the effects of chemicals, 2 × 10^4^ cells (KB cells) or 2 × 10^5^ cells (HepG2 cells) were seeded in each well of a 24-well culture plate. After seeding (24 h later), cells were treated with cardiac glycoside (ouabain, oleandrin or digitoxin) or JPH203 for 24 h, and the cell numbers in each well were counted. To check the effects of siRNAs, KB cells (2 × 10^4^ cells) were transfected with siNC or siTHADA, and the medium was changed 5 h after transfection. Then, the cell numbers in each well were counted 24 h, 48 h, and 72 h after transfection. When indicated, the cells transfected with siTHADA for 24 h were further treated with human THADA-expressing vector (hTHADA) or empty vector (mock) using Lipofectamine 3000, and the medium was changed 5 h after treatment.

### Preparation of the lysate samples from the cells

To prepare the lysate samples, the cells cultured in 12-well or 24-well plate were washed once with ice-cold phosphate buffered saline (PBS) and treated with lysis buffer (150 mM NaCl, 50 mM Tris–HCl (pH 7.4), 1 mM EDTA, and 1% Triton X-100) on ice for 20 min. Then, the cells were centrifuged at 16,000×*g* for 20 min at 4 °C, and the supernatant was collected. The protein concentration of the samples was quantified by the absorbance at 570 nm using BCA Protein Assay Kit and bovine serum albumin (BSA) as a standard.

### Western blotting

Western blotting was carried out as described previously [12]. As primary antibodies, anti-THADA (1:5000 dilution), anti-4F2hc (1:2500 dilution), anti-β-actin (1:5000 dilution), anti-Na^+^,K^+^-ATPase α1-isoform (1:2500 dilution), and anti-LAT1 (1:1000 dilution) antibodies were used. As secondary antibodies, horseradish peroxidase-conjugated anti-rabbit and anti-mouse IgG antibodies were used (1:5000 dilution). Chemiluminescence was observed using Western Lighting ECL Pro and detection was performed by LAS-4000 plus system (Fujifilm).

### Immunocytochemistry

KB cells cultured on coverslips were fixed in ice-cold methanol for 5 min and permeabilized with PBS containing 0.1 mM CaCl_2_, 1 mM MgCl_2_, 0.3% Triton X-100 and 0.1% BSA for 15 min at room temperature. For blocking, the goat serum dilution buffer (GSDB; PBS supplemented with 300 mM NaCl, 17% goat serum and 0.3% Triton X-100) was used. The cells were treated with the anti-THADA (1:100 dilution), anti-β-actin (1:50 dilution), anti-Na^+^,K^+^-ATPase α1-isoform (1:100 dilution) or anti-Na^+^,K^+^-ATPase α3-isoform (1:100 dilution) antibody overnight at 4 °C and then with Alexa Fluor 488-conjugated anti-rabbit IgG and Alexa Fluor 546-conjugated anti-mouse IgG antibodies (1:100 dilution) for 60 min at room temperature. DNA was visualized using DAPI (1:1000 dilution). Immunofluorescence images were visualized by using a Zeiss LSM 780 laser scanning confocal microscope.

### Statistical analysis

Results are shown as means ± standard error of the mean. Differences between groups were analyzed by one way analysis of variance, and correction for multiple comparisons was made by using Tukey’s multiple comparison test. Comparison between the two groups was made by using Student’s t test. Statistically significant differences were assumed at P < 0.05.

## Results

### Inhibition of the THADA expression by cardiac glycosides

**Fig. 1:**
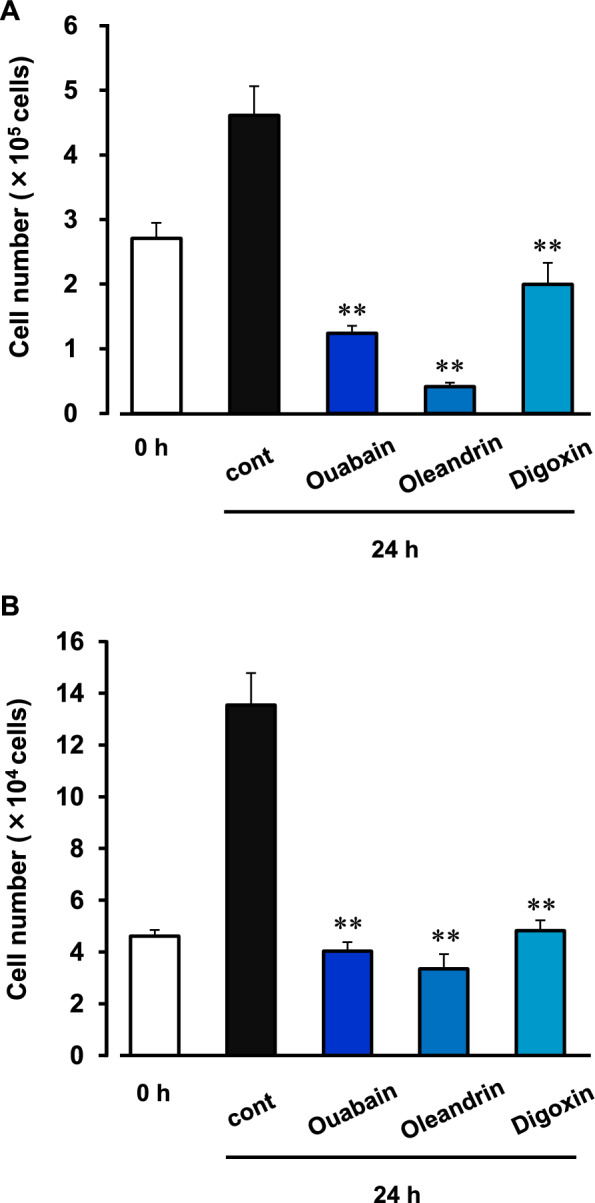
Inhibition of cancer cell proliferation by cardiac glycosides. HepG2 cells (**A**) and KB cells (**B**) were treated with 300 nM ouabain, oleandrin, or digoxin for 24 h. The cell number was counted before (0 h) and after (24 h) treatments of cardiac glycosides. As a control, the cells were cultured in the absence of cardiac glycosides for 24 h (cont). n = 3–6. **P < 0.01 versus control

**Fig. 2:**
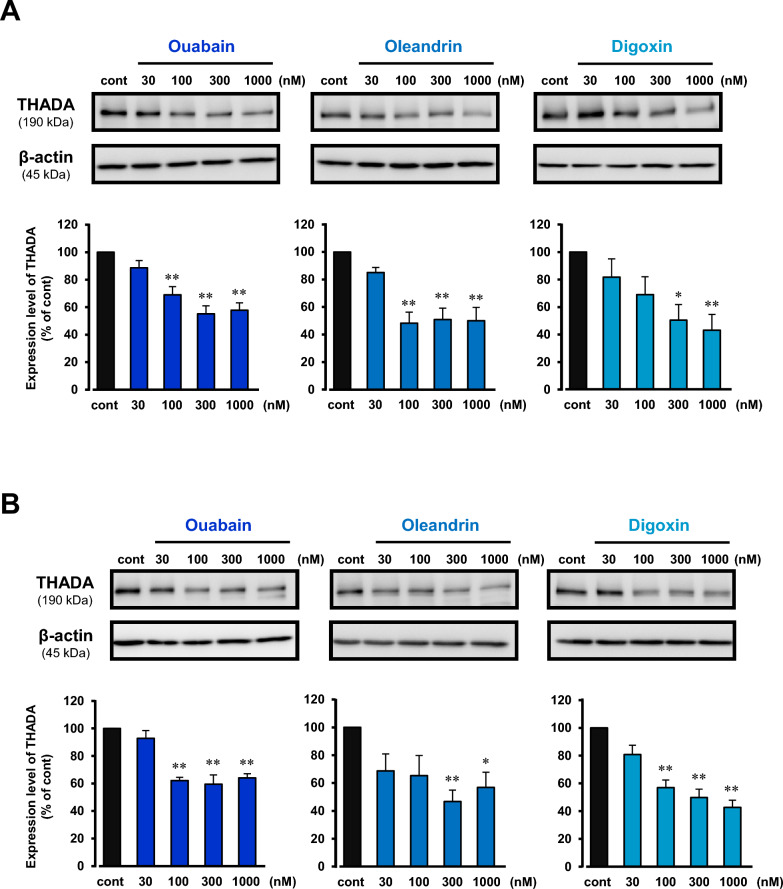
Inhibition of expression of THADA protein by cardiac glycosides. HepG2 cells (**A**) and KB cells (**B**) were treated with and without 30–1000 nM ouabain (left), oleandrin (middle) or digoxin (right) for 24 h. As a control, the cells were cultured in the absence of cardiac glycosides for 24 h (cont). Then, the lysate samples (15 μg/lane) were prepared, and Western blotting was performed. Typical images of the blotting with anti-THADA and anti-β-actin antibodies were shown in upper panels. Expression level of THADA (190 kDa) was normalized by corresponding β-actin expression (45 kDa), and the quantitative data were shown in lower panels. n = 4–6. *P < 0.05 and **P < 0.01 versus control (cont)

In the present study, three kinds of cardiac glycosides (ouabain, oleandrin, and digoxin) were used. All of them at 300 nM significantly inhibited the cell proliferation in HepG2 and KB cells (Fig. 1). To clarify the molecules involved in the cardiac glycosides-elicited pathway, we performed microarray analysis using HepG2 cells treated with or without ouabain (1 µM). In the analysis, the expression of THADA was found to be markedly decreased in the ouabain-treated cells (Additional file 1: Fig. S1A). In fact, ouabain (1 µM) decreased the expression of THADA mRNA in the cells (Additional file 1: Fig. S1B). In HepG2 and KB cells, ouabain, oleandrin, and digoxin (30 nM–1 µM) decreased the expression level of THADA protein in a concentration-dependent manner (Fig. 2).

### Inhibition of cancer cell proliferation by THADA-knockdown

**Fig. 3:**
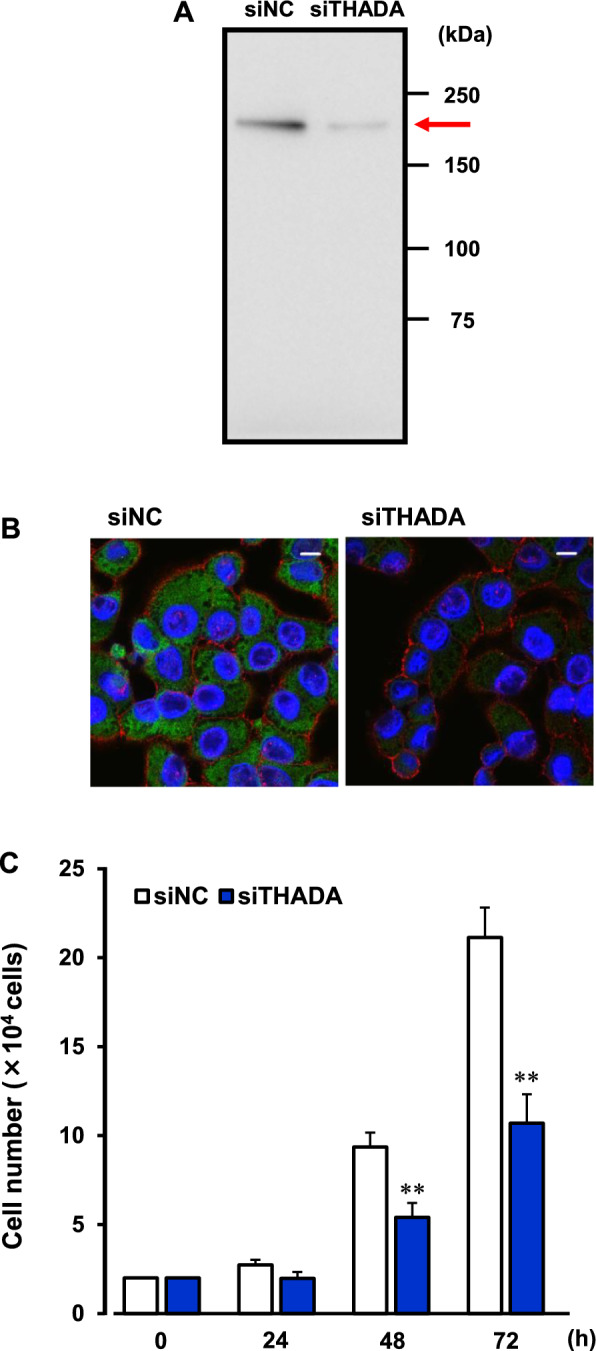
Inhibition of cancer cell proliferation by THADA-knockdown. **A** Expression of THADA protein (190 kDa) in KB cells treated with THADA siRNA (siTHADA) or negative control siRNA (siNC) for 48 h. Western blotting was performed by using the lysate samples (15 μg/lane) and anti-THADA antibody. Typical image of the blotting was shown. **B** Immunocytochemistry of THADA in KB cells treated with siTHADA or siNC or 48 h. Localization of THADA (green) and β-actin (red) was shown. DAPI was used for staining nucleus (blue). Scale bars, 10 μm. **C** KB cells were treated with siTHADA or siNC. The siTHADA- or siNC-transfected cells were seeded (2 × 10^4^ cells; 0 h). Then, the cell number was couted 24, 48, and 72 h after the treatment. n = 9. **P < 0.01 versus siNC

To examine properties of THADA protein in the cancer cells, THADA was knocked down by using siRNA in HepG2 and KB cells. The cells were transfected with THADA siRNA (siTHADA) or negative control siRNA (siNC). The transfection efficiency into these cells was 8.7 ± 2.3% (HepG2; n = 5) and 83.7 ± 4.3% (KB; n = 5). Actually, the expression level of THADA protein was markedly decreased by siTHADA in KB cells (Fig. 3A, B) but not in HepG2 cells (data not shown). Thus, we used KB cells to assess the pathophysiological properties of THADA in the following experiments.

**Fig. 4:**
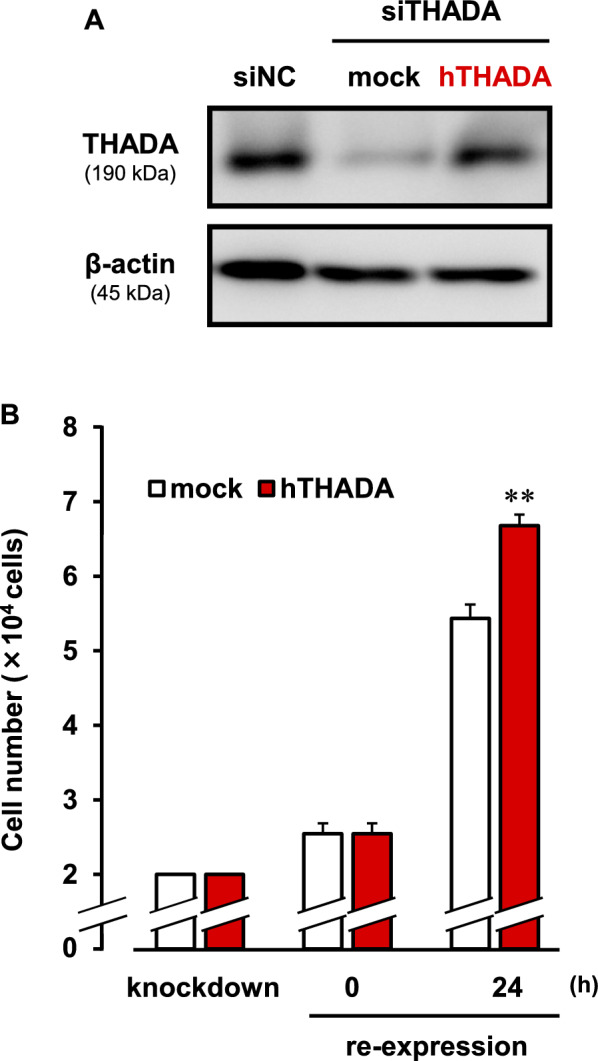
Re-expression of THADA in the THADA-knockdown cells. **A**, **B** The KB cells transfected the THADA siRNA (siTHADA) for 24 h were further treated with human THADA-expressing vector (hTHADA) or empty vector (mock), and the medium was changed 5 h after treatment. In **A**, the cells were subsequently cultured for 24 h, and the lysate samples were prepared. Western blotting of the samples (7.5 μg/lane) was performed with anti-THADA and anti-β-actin antibodies. As a control, the lysate sample (7.5 μg/lane) from KB cells treated with negative control siRNA (siNC) for 48 h was used. Typical images of the blotting were shown. In **B**, the siTHADA-transfected cells were seeded (2 × 10^4^ cells; knockdown). Then, the cell numbers of hTHADA- or mock-transfected cells were counted 0 h and 24 h after the subsequent culture. n = 8. **P < 0.01 versus mock

In the KB cells, the cell proliferation was inhibited by the treatment of siTHADA (Fig. 3C). After the knockdown of THADA, we tried to rescue the THADA expression in the KB cells (Fig. 4A). The expression level of THADA in the rescued cells was comparable to that in the siNC-transfected cells (Fig. 4A). Interestingly, the cell proliferation was significantly stimulated by re-expression of THADA in the THADA-knockdown cells (Fig. 4B). These results suggest that negative regulation of THADA expression is involved in the cardiac glycosides-induced anti-cancer effect.

### Down-regulation of SLC7A5 (LAT1) and SLC3A2 (4F2hc) in the THADA-knockdown cells

**Fig. 5:**
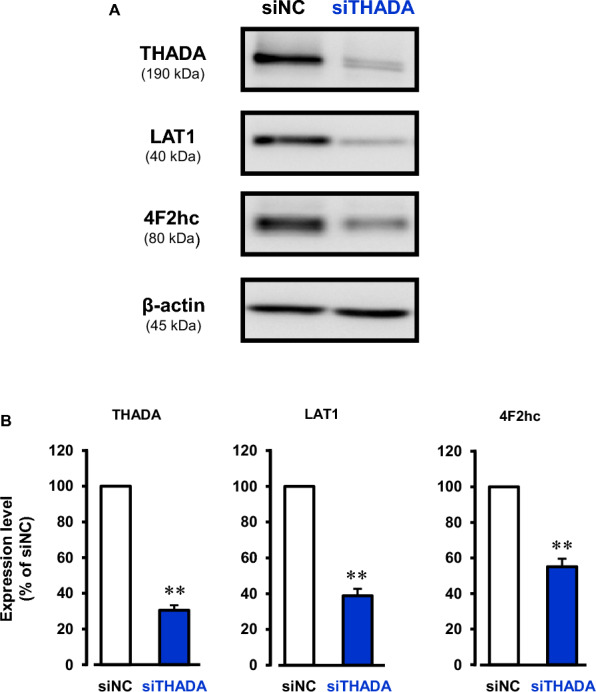
Decrease in expression of LAT1 and 4F2hc proteins by treatment of THADA siRNA (siTHADA). **A** Expression of THADA (190 kDa), LAT1 (40 kDa), 4F2hc (80 kDa), and β-actin (45 kDa) in KB cells. The cells were treated with siTHADA or negative control siRNA (siNC) for 48 h, and then the lysate samples were prepared. Western blotting of the samples (15 μg/lane) was performed. Typical images of the blotting were shown. **B** Expression levels of THADA, LAT1, and 4F2hc were normalized by corresponding β-actin expression, and the quantitative data were shown. n = 8–9. **P < 0.01 versus siNC

To find the genes affected by the THADA-knockdown, microarray analysis was performed. We used KB cells transfected with siTHADA or siNC. Here, we focused on the change in expression of the solute carrier (SLC) transporters involved in nutrient uptake in cancer cells [13, 14], and found that the expression levels of SLC7A5 (LAT1; L-type amino acid transporter 1) and SLC3A2 (4F2hc; 4F2 heavy chain) were markedly decreased by the THADA-knockdown (Additional file 1: Fig. S2). LAT1, a Na^+^-independent amino acid transporter [15], is highly expressed in many types of cancer cells and associated with cancer cell proliferation [16]. LAT1 forms the hetero dimer with 4F2hc, an essential protein for functional expression of LAT1 [15]. In Western blotting, expression of both LAT1 and 4F2hc proteins was significantly decreased by the THADA-knockdown in KB cells (Fig. 5).

### Negative regulation of THADA-LAT1 pathway in the cardiac glycosides-induced inhibition of cancer cell proliferation

Next, we examined the effects of cardiac glycosides on the expression of LAT1 and 4F2hc in KB cells. Similar to the THADA-knockdown cells (Additional file 1: Fig. S2), LAT1 and 4F2hc mRNAs were markedly decreased in the ouabain (100 nM)-treated KB cells, and no significant change in the mRNA expression of SLC2A13, SLC12A7, and SLC39A9 was observed (Additional file 1: Fig. S3). It is noted that SLC7A11 mRNA was slightly but significantly decreased by ouabain (Additional file 1: Fig. S3).

**Fig. 6:**
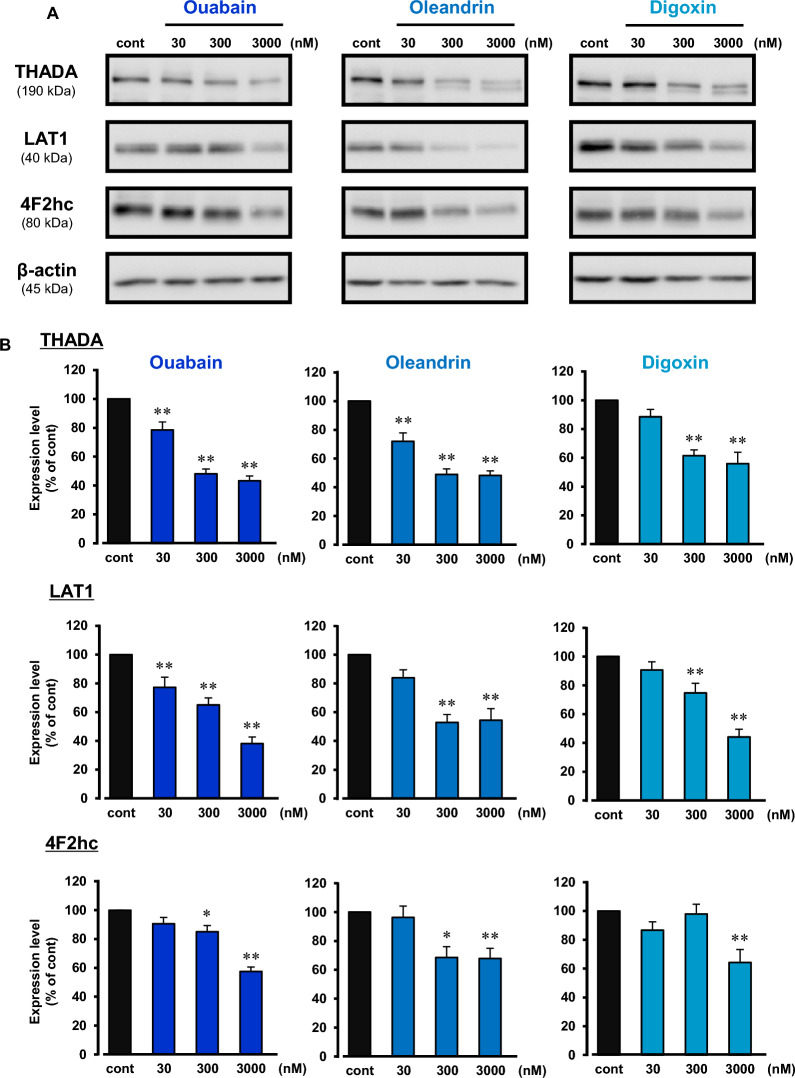
Decrease in expression of LAT1 and 4F2hc proteins by cardiac glycosides. **A** Expression of THADA (190 kDa), LAT1 (40 kDa), 4F2hc (80 kDa), and β-actin (45 kDa) in KB cells. The cells were treated with 30–3000 nM ouabain (left), oleandrin (middle), or digoxin (right) for 24 h, and then the lysate samples were prepared. As a control, the cells were cultured in the absence of cardiac glycosides for 24 h (cont). Western blotting of the samples (15 μg/lane) was performed, and typical images of the blotting were shown. **B** Expression levels of THADA, LAT1, and 4F2hc were normalized by corresponding β-actin expression, and the quantitative data were shown. n = 5–16. *P < 0.05 and **P < 0.01 versus control (cont)

Three kinds of cardiac glycosides (ouabain, oleandrin, and digoxin) inhibited the expression of THADA protein in a concentration-dependent manner (30 nM–3 µM) in KB cells (Fig. 6). These compounds also decreased the protein expression levels of LAT1 and 4F2hc in a concentration-dependent manner (30 nM–3 µM) (Fig. 6). JPH203 (KYT-0353), a selective inhibitor of LAT1 [17], significantly inhibited cell proliferation of KB cells (Additional file 1: Fig. S4). These results suggest that THADA-LAT1 pathway is associated with the cardiac glycosides-induced inhibition of cancer cell proliferation.

### Colocalization of THADA with intracellular Na^+^,K^+^-ATPase α3-isoform

In KB cells, Na^+^,K^+^-ATPase α1-isoform (α1NaK) was expressed in the plasma membrane (Additional file 1: Fig. S5A), while Na^+^,K^+^-ATPase α3-isoform (α3NaK) was in the cytoplasm (Additional file 1: Fig. S5B), as well as the case in human colorectal cancer HT-29 cells, gastric cancer MKN45 cells, and hepatocellular carcinoma HepG2 cells [18]. Interestingly, THADA was partially colocalized with α3NaK but not α1NaK in KB cells (Fig. S5C).

## Discussion

Cardiac glycosides, such as digoxin and digitoxin, have been used for treating congestive heart failure and cardiac arrhythmia by selective inhibition of Na^+^,K^+^-ATPase. On the other hand, many reports suggest that cardiac glycosides may be potential chemicals for cancer, because they inhibit cell proliferation through multiple pathways, including activation of Src/MAPK signaling, induction of intracellular Ca^2+^ signaling, and inhibition of PI3K/AKT/mTOR, HIF-1, and Wnt/β-catenin signaling [19–21]. In this study, we revealed that downregulation of the THADA-LAT1 pathway is involved in the cardiac glycoside-induced anti-cancer effect. Three different inhibitors of Na^+^,K^+^-ATPase (cardiac glycosides such as ouabain, oleandrin, and digoxin) inhibited the proliferation of human cancer cells (KB and HepG2 cells), and markedly decreased the expression levels of THADA, LAT1, and 4F2hc in the cells. It has been reported that LAT1 is specifically expressed in the plasma membrane of cancer cells and contributes to massive uptake of amino acids [16]. Here, a selective inhibitor of LAT1 (JPH203) inhibited the cancer cell proliferation. Therefore, a decrease in the amino acid uptake by the THADA-mediated negative regulation of LAT1 is at least partially involved in the cardiac glycoside-induced effects. In addition, cystine/glutamate antiporter xCT (SLC7A11) may be involved in this mechanism, because 4F2hc regulates the stability and transport activity of it in cancer cells [22]. In fact, the xCT mRNA was decreased in the ouabain-treated KB cells (Additional file 1: Fig. S3). Thus, cystine uptake into cancer cells via xCT may be impaired in the cancer cells treated with cardiac glycosides.

So far, it has been reported that THADA is colocalized with ER Ca^2+^-ATPase (SERCA2), which belongs to P2-type ATPase family [6], in pancreatic β-cells, and that the THADA-SERCA2 association contributes to impairment of insulin secretion [5]. In the present study, we found colocalization of THADA with Na^+^,K^+^-ATPase α3-isoform (α3NaK), which is also involved in P2-type ATPase family [6]. In various types of human cancer cells, α3NaK is found to be aberrantly expressed in intracellular vesicles in which Rab10, a small GTPase, is localized [18]. Recently, we found that cardiac glycosides (ouabain, oleandrin, and digoxin) stimulate glucose transporter GLUT1 endocytosis in HepG2 and KB cells and inhibit glycolysis of the cells by targeting the intracellular α3NaK [21]. In the cells, the [^3^H]-ouabain uptake was increased in a time- and temperature-dependent manner and reached a maximum at 10 min [18, 21]. Interestingly, a low concentration of ouabain (0.2 μM) significantly inhibited the enzyme activity of α3NaK but not α1NaK in the cancer cells [21]. Here, we found that significant effects of ouabain on the expression levels of THADA and LAT1 were observed at around 0.1 μM. These results suggest that cardiac glycosides act on intracellular α3NaK that may be functionally associated with THADA. In future studies, it is necessary to clarify the mechanism of action of cardiac glycosides by using the THADA-, LAT1-, or 4F2hc-overexpressing cancer cells.

Li et al. [8] reported that THADA has a critical role in Golgi residency of programmed death-ligand 1 (PD-L1) and upregulates the expression of PD-L1 in human colorectal cancer cells. In this mechanism, THADA is associated with PD-L1 in the Sec24A-dependent coat protein complex II (COPII) vesicles. As the upregulation of PD-L1 in cancer cells inhibits T cell-mediated cytotoxicity, THADA is suggested to be a promising target for overcoming PD-L1-dependent immune evasion [8]. Our results of cardiac glycosides-induced downregulation of THADA may be effective in decreasing the THADA-PD-L1 interaction in cancer cells.

Epidemiological data showed that patients who have received digitalis therapy are more protected from some types of malignancies such as breast, lymphoma/leukemia, and prostate/urinary cancers [23–26]. Menger et al. [27] reported that cardiac glycosides significantly enhance overall survival in cancer patients by using a text-based research algorithm to identify all cancer patients who received cardiac glycosides during conventional cancer therapies (between 1981 and 2009). Our present findings may explain one of the mechanisms of cardiac glycoside-induced anti-cancer effects.

## Conclusions

In the present study, we found that negative regulation of the expression of THADA and LAT1 is related to the anti-proliferative mechanisms induced by cardiac glycosides. We suggest that binding of cardiac glycosides to intracellular Na^+^,K^+^-ATPase α3-isoform negatively regulates the THADA-LAT1 pathway and subsequent cancer cell proliferation. This study provides the basis for developing the cardiac glycoside-relating drugs to inhibit the THADA expression for treatment of cancers with clinical benefit.

### Supplementary Information

**Additional file 1: Fig. S1.** Decrease in expression of THADA mRNA by ouabain. **A** Change in expression of THADA in HepG2 cells treated with ouabain (1 μM for 12 h). In microarray gene expression analysis, the expression of THADA was assessed by two probes. The values indicate negative fold change of ouaban-treated samples compared with the ouabain-untreated cells. **B** RT-PCR images of THADA and GAPDH in HepG2 cells. Cells were cultured in the presence and absence of ouabain (1 μM) for 24 h. **Fig. S2.** Change in expression of SLC transporters involved in nutrient uptake by THADA-knockdown. KB cells were treated with THADA siRNA (siTHADA) or negative control siRNA (siNC) for 72 h, and then total RNA samples were prepared. Microarray gene expression analysis was performed using these samples. In the graph, relative expression levels of the SLC transporters (siTHADA/siNC) were shown. **Fig. S3.** Change in expression of SLC transporters (picked up from Fig. S2) in the ouabain-treated KB cells. KB cells were treated with or without (control) ouabain (100 nM) for 48 h, and then total RNA samples were prepared. Real-time PCR was performed using these samples. Expression levels of SLC transporters were normalized by corresponding GAPDH expression, and the quantitative data were shown. n = 3–4. *P < 0.05 and **P < 0.01 versus control cells. **Fig. S4.** Inhibition of cancer cell proliferation by JPH203, a LAT1 inhibitor. KB cells (3 × 10^4^ cells) were treated with 3–100 μM JPH203 for 24 h. As a control, the cells were cultured without JPH203 (cont). The cell number was counted just after (0 h) and 24 h after the treatment. n = 3. *P < 0.05 and **P < 0.01 versus control (cont). **Fig. S5.** Localization of Na^+^,K^+^-ATPases and THADA in KB cells. **A** Immunocytochemistry using anti-Na^+^,K^+^-ATPase α1-isoform (α1NaK) (red) and anti-THADA (green) antibodies in the cells. DNA in the nucleus was visualized with DAPI (blue). Scale bars, 10 µm. **B** Immunocytochemistry using anti-Na^+^,K^+^-ATPase α3-isoform (α3NaK) (red) and anti-THADA (green) antibodies in the cells. DNA in the nucleus was visualized with DAPI (blue). Scale bars, 10 µm. **C** Colocalization coefficient of THADA with α1NaK or α3NaK was calculated with Pearson correlation coefficient analysis. n = 11–13.

## Data Availability

All the data and materials are available.

## Abbreviations

α1NaK: Na^+^,K^+^-ATPase α1-isoform
α3NaK: Na^+^,K^+^-ATPase α3-isoform
DAPI: 4′,6-Diamidino-2-phenylindole
4F2hc: 4F2 heavy chain
LAT1: L-type amino acid transporter 1
PD-L1: Programmed death-ligand 1
SERCA: Sarco/ER Ca^2+^-ATPase
THADA: Thyroid adenoma-associated protein
xCT: Cystine/glutamate antiporter
