# Supplementary material for: Negative regulation of thyroid adenoma-associated protein (THADA) in the cardiac glycoside-induced anti-cancer effect
Source: J Physiol Sci. 2024 Apr 1;74:23. doi: 10.1186/s12576-024-00914-7 (PMC10985892; doi:10.1186/s12576-024-00914-7)
Supplement: Supplementary file 1 — Additional file 1: Fig. S1. Decrease in expression of THADA mRNA by ouabain. A Change in expression of THADA in HepG2 cells treated with ouabain (1 μM for 12 h). In microarray gene expression analysis, the expression of THADA was assessed by two probes. The values indicate negative fold change of ouaban-treated samples compared with the ouabain-untreated cells. B RT-PCR images of THADA and GAPDH in HepG2 cells. Cells were cultured in the presence and absence of ouabain (1 μM) for 24 h. Fig. S2. Change in expression of SLC transporters involved in nutrient uptake by THADA-knockdown. KB cells were treated with THADA siRNA (siTHADA) or negative control siRNA (siNC) for 72 h, and then total RNA samples were prepared. Microarray gene expression analysis was performed using these samples. In the graph, relative expression levels of the SLC transporters (siTHADA/siNC) were shown. Fig. S3. Change in expression of SLC transporters (picked up from Fig. S2) in the ouabain-treated KB cells. KB cells were treated with or without (control) ouabain (100 nM) for 48 h, and then total RNA samples were prepared. Real-time PCR was performed using these samples. Expression levels of SLC transporters were normalized by corresponding GAPDH expression, and the quantitative data were shown. n = 3–4. *P < 0.05 and **P < 0.01 versus control cells. Fig. S4. Inhibition of cancer cell proliferation by JPH203, a LAT1 inhibitor. KB cells (3 × 10^4^ cells) were treated with 3–100 μM JPH203 for 24 h. As a control, the cells were cultured without JPH203 (cont). The cell number was counted just after (0 h) and 24 h after the treatment. n = 3. *P < 0.05 and **P < 0.01 versus control (cont). Fig. S5. Localization of Na+,K+-ATPases and THADA in KB cells. A Immunocytochemistry using anti-Na+,K+-ATPase α1-isoform (α1NaK) (red) and anti-THADA (green) antibodies in the cells. DNA in the nucleus was visualized with DAPI (blue). Scale bars, 10 µm. B Immunocytochemistry using anti-Na+,K+-ATPase α3-isoform (α3NaK) [file 12576_2024_914_MOESM1_ESM.pdf]

Figure S1

A

	Ouabain #1	Ouabain #2
THADA probe 1	-9.188	-6.852
THADA probe 2	-47.110	-7.035

B

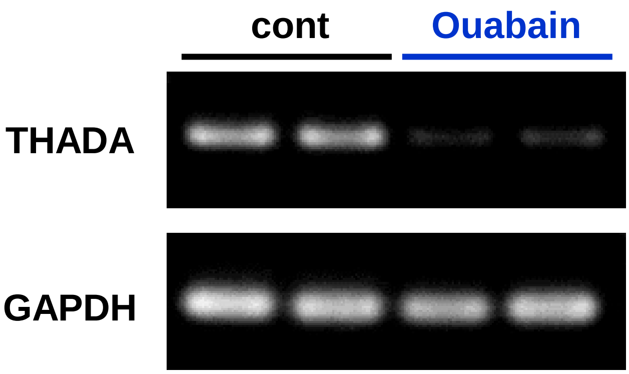

Fig. S1 Decrease in expression of THADA mRNA by ouabain. A Change in expression of THADA in HepG2 cells treated with ouabain (1 μM for 12 h). In microarray gene expression analysis, the expression of THADA was assessed by two probes. The values indicate negative fold change of ouaban-treated samples compared with the ouabain-untreated cells. B RT-PCR images of THADA and GAPDH in HepG2 cells. Cells were cultured in the presence and absence of ouabain (1 μM) for 24 h.

Figure S2

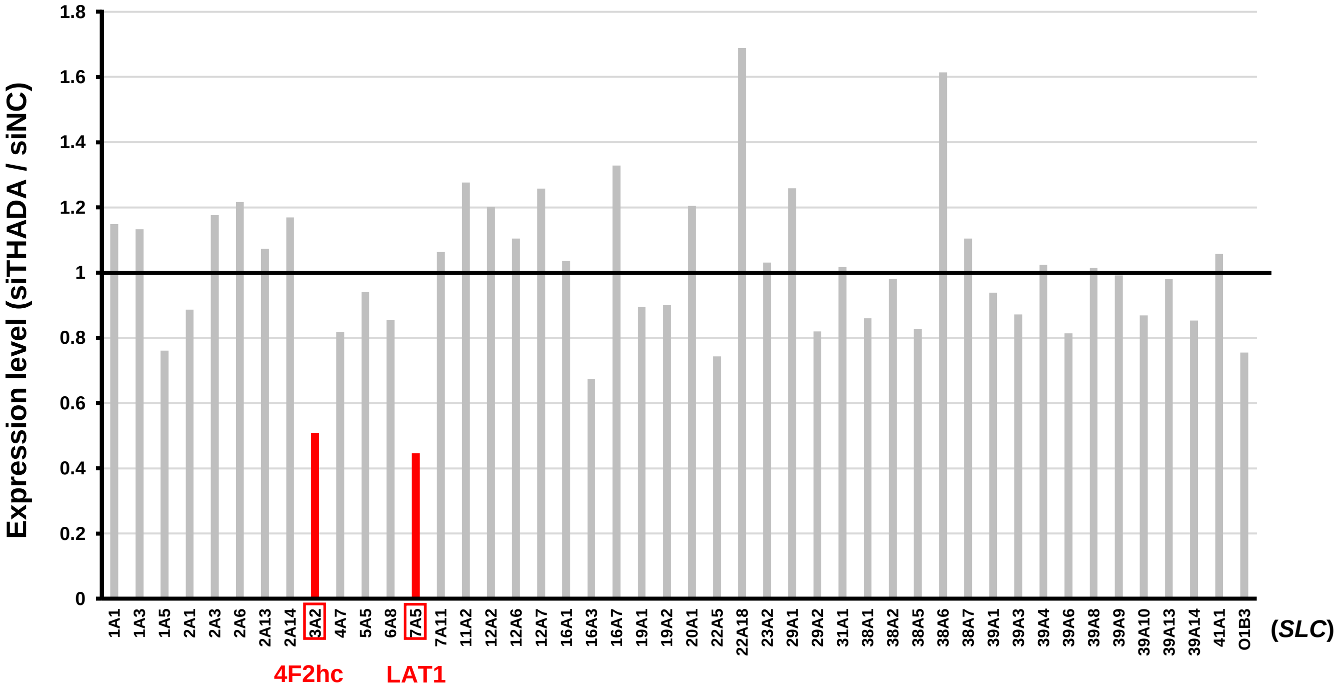

Fig. S2 Change in expression of SLC transporters involved in nutrient uptake by THADA-knockdown. KB cells were treated with THADA siRNA (siTHADA) or negative control siRNA (siNC) for 72 h, and then total RNA samples were prepared. Microarray gene expression analysis was performed using these samples. In the graph, relative expression levels of the SLC transporters (siTHADA/siNC) were shown.

Figure S3

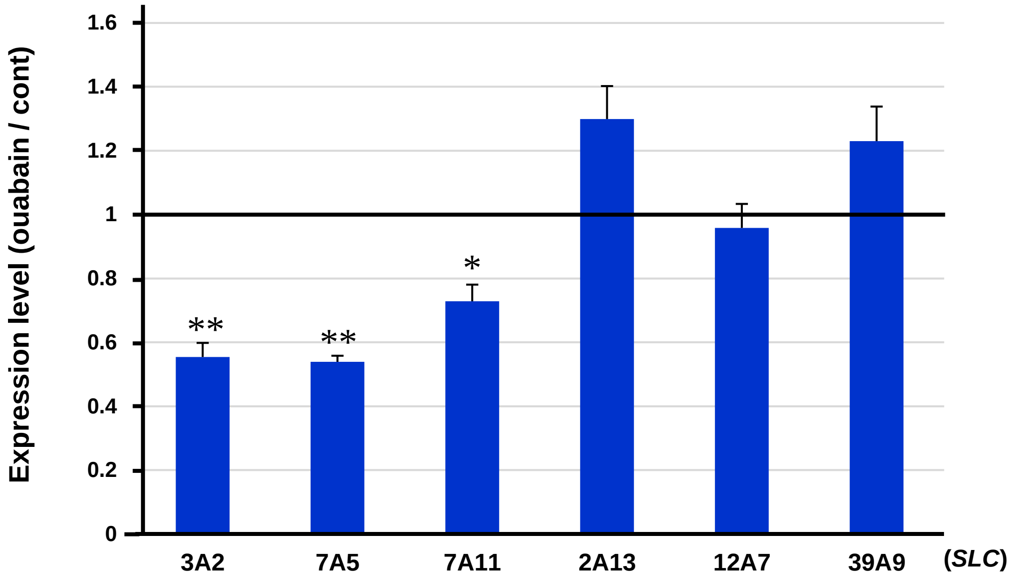

Fig. S3 Change in expression of SLC transporters (picked up from Fig. S2) in the ouabain-treated KB cells. KB cells were treated with or without (control) ouabain (100 nM) for 48 h, and then total RNA samples were prepared. Real-time PCR was performed using these samples. Expression levels of SLC transporters were normalized by corresponding GAPDH expression, and the quantitative data were shown. n = 3-4. *P < 0.05 and **P < 0.01 versus control cells.

Figure S4

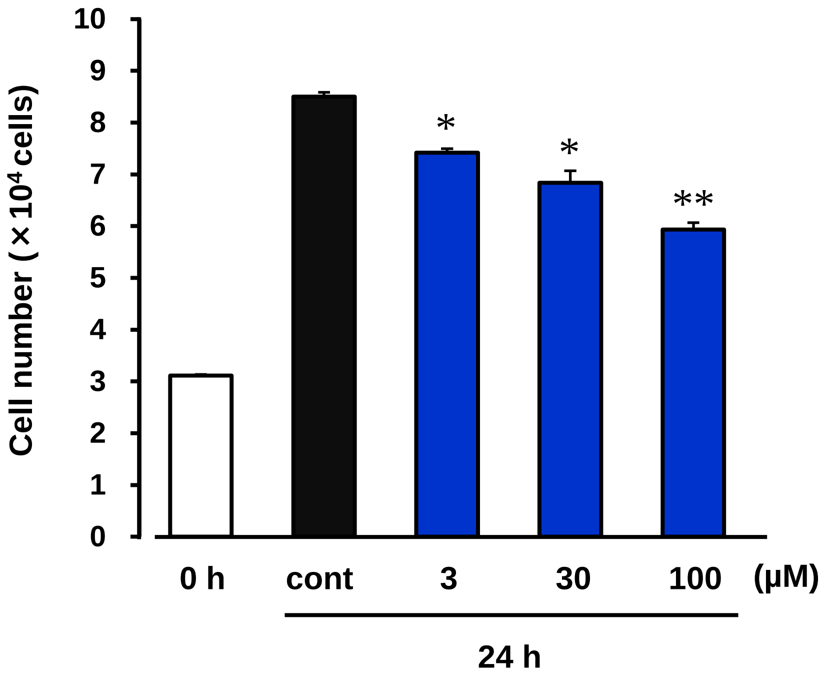

Fig. S4 Inhibition of cancer cell proliferation by JPH203, a LAT1 inhibitor. KB cells (3 x 104 cells) were treated with 3-100 μM JPH203 for 24 h. As a control, the cells were cultured without JPH203 (cont). The cell number was counted just after (0 h) and 24 h after the treatment. n = 3. *P < 0.05 and **P < 0.01 versus control (cont).

Figure S5

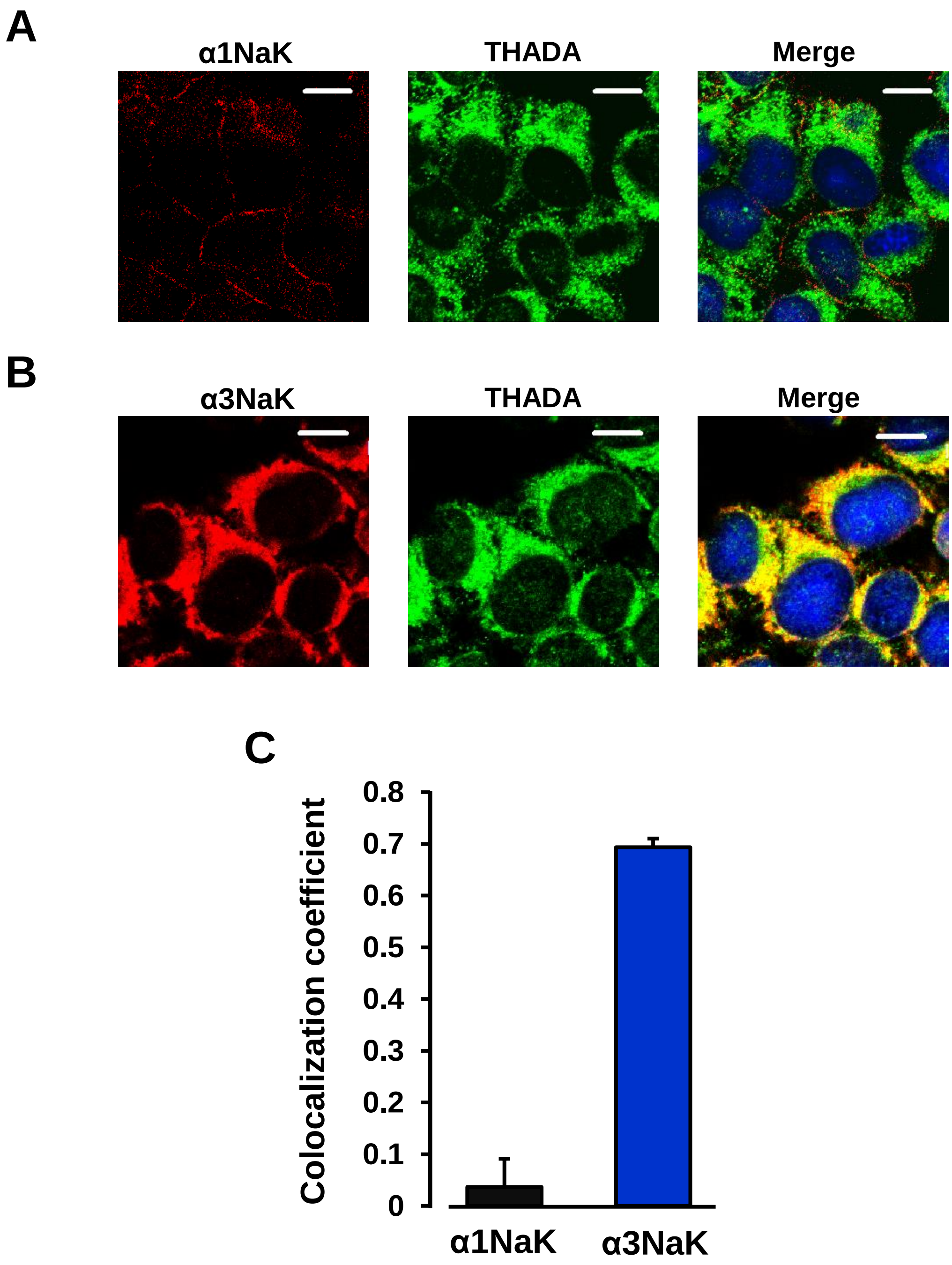

Fig. S5 Localization of Na^+,K^+-ATPases and THADA in KB cells. A Immunocytochemistry using anti-Na^+,K^+-ATPase α1-isoform (α1NaK) (red) and anti-THADA (green) antibodies in the cells. DNA in the nucleus was visualized with DAPI (blue). Scale bars, 10 µm.

B Immunocytochemistry using anti-Na^+,K^+-ATPase α3-isoform (α3NaK) (red) and anti-THADA (green) antibodies in the cells. DNA in the nucleus was visualized with DAPI (blue). Scale bars, 10 µm. C Colocalization coefficient of THADA with α1NaK or α3NaK was calculated with Pearson correlation coefficient analysis. n = 11-13.
